# Supplementary material for: Wave-Encoded Model-Based Deep Learning for Highly Accelerated Imaging with Joint Reconstruction
Source: Bioengineering (Basel). 2022 Nov 29;9(12):736. doi: 10.3390/bioengineering9120736 (PMC9774601; doi:10.3390/bioengineering9120736)
Supplement: Supplementary file 1 [file bioengineering-09-00736-s001.zip › bioengineering-1988151-supplementary.pdf]

Supplementary Information

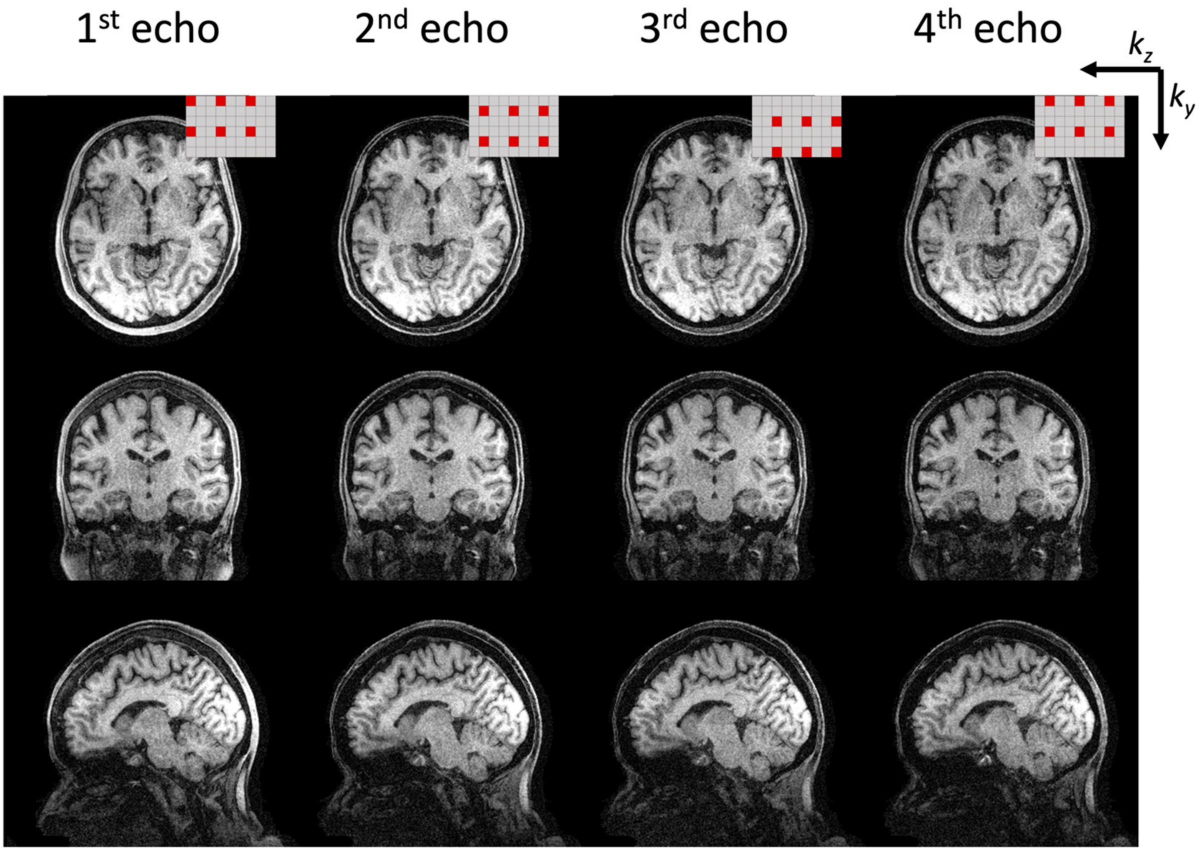

Figure S1. Each echo images of wave-MoDL at R=3x3-fold acceleration.

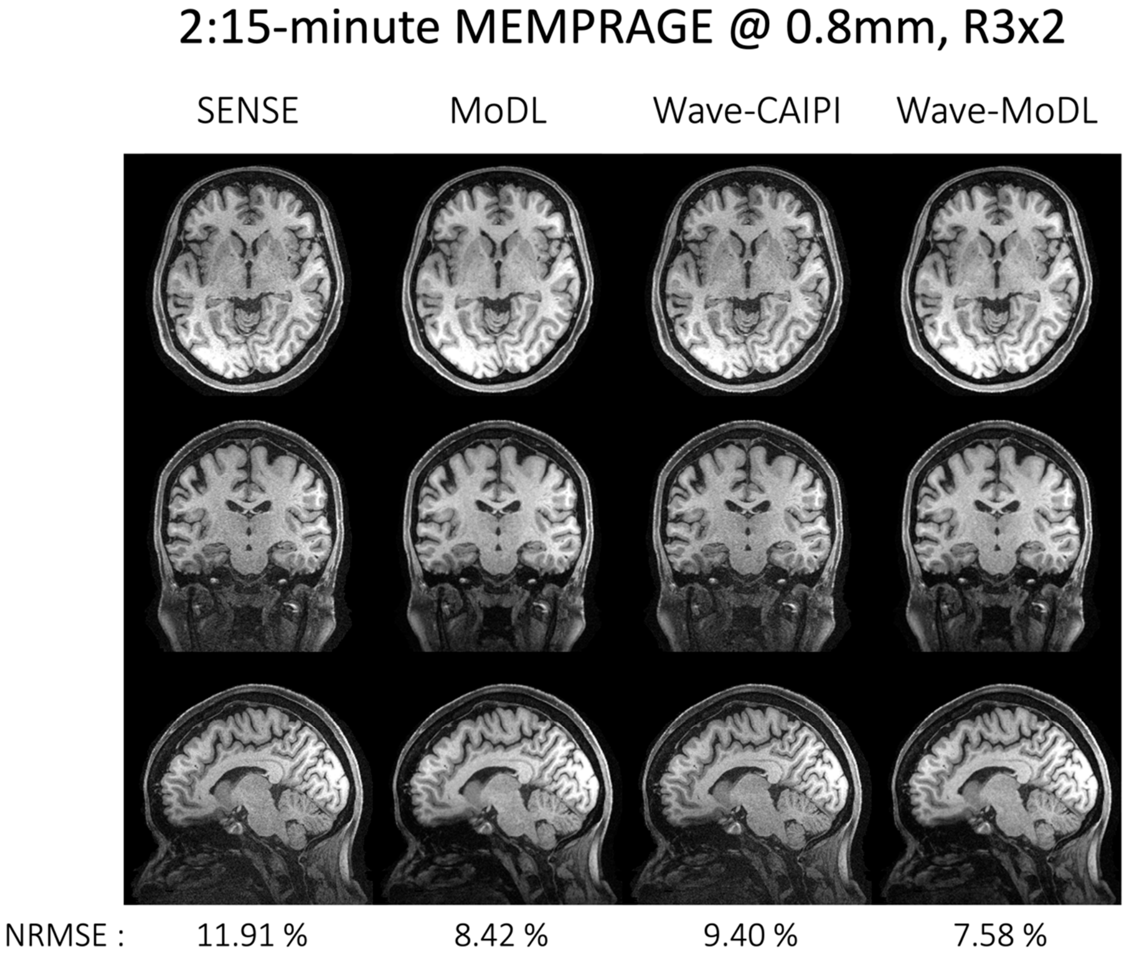

Figure S2. The proposed method on the MEMPRAGE database at R=3x2-fold and 0.8 mm isotropic voxel resolution. Echo images were combined with root mean squared. NRMSEs were calculated for the entire testing database.

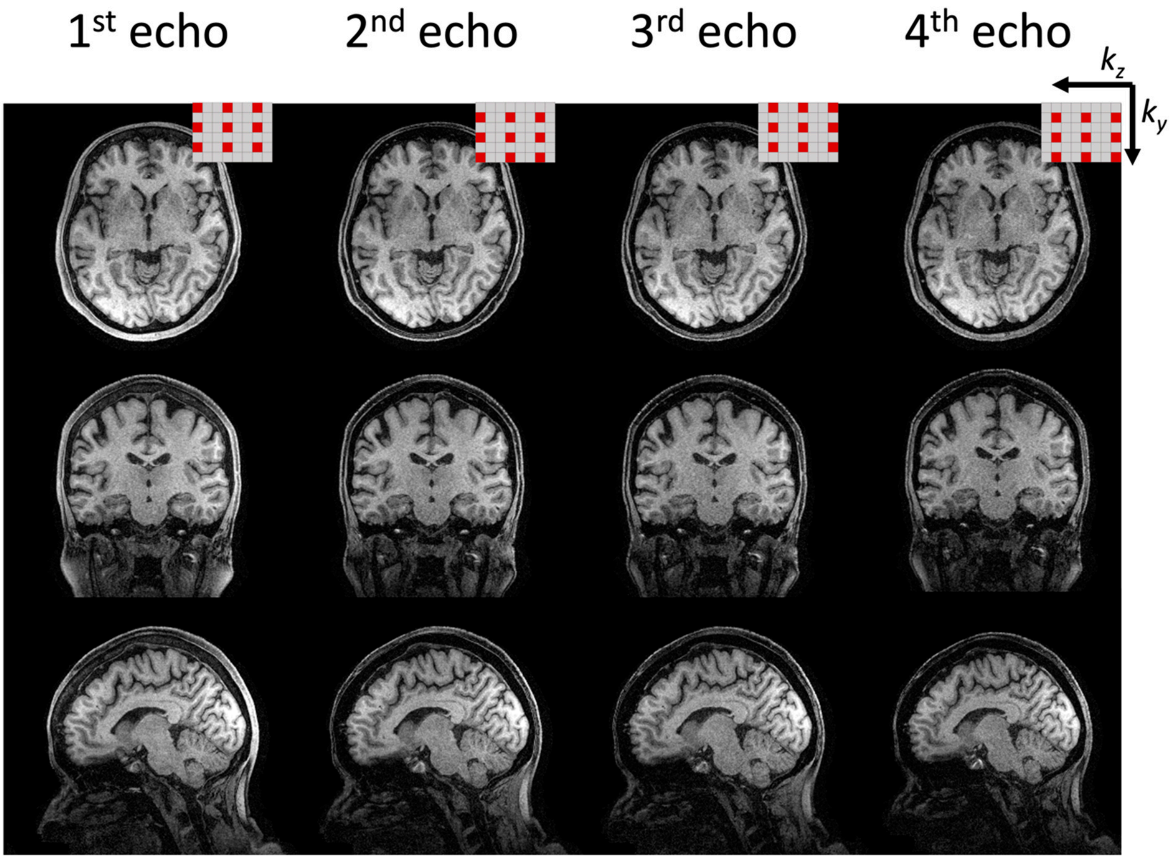

Figure S3. Each echo image of wave-MoDL at R=3x2-fold acceleration.

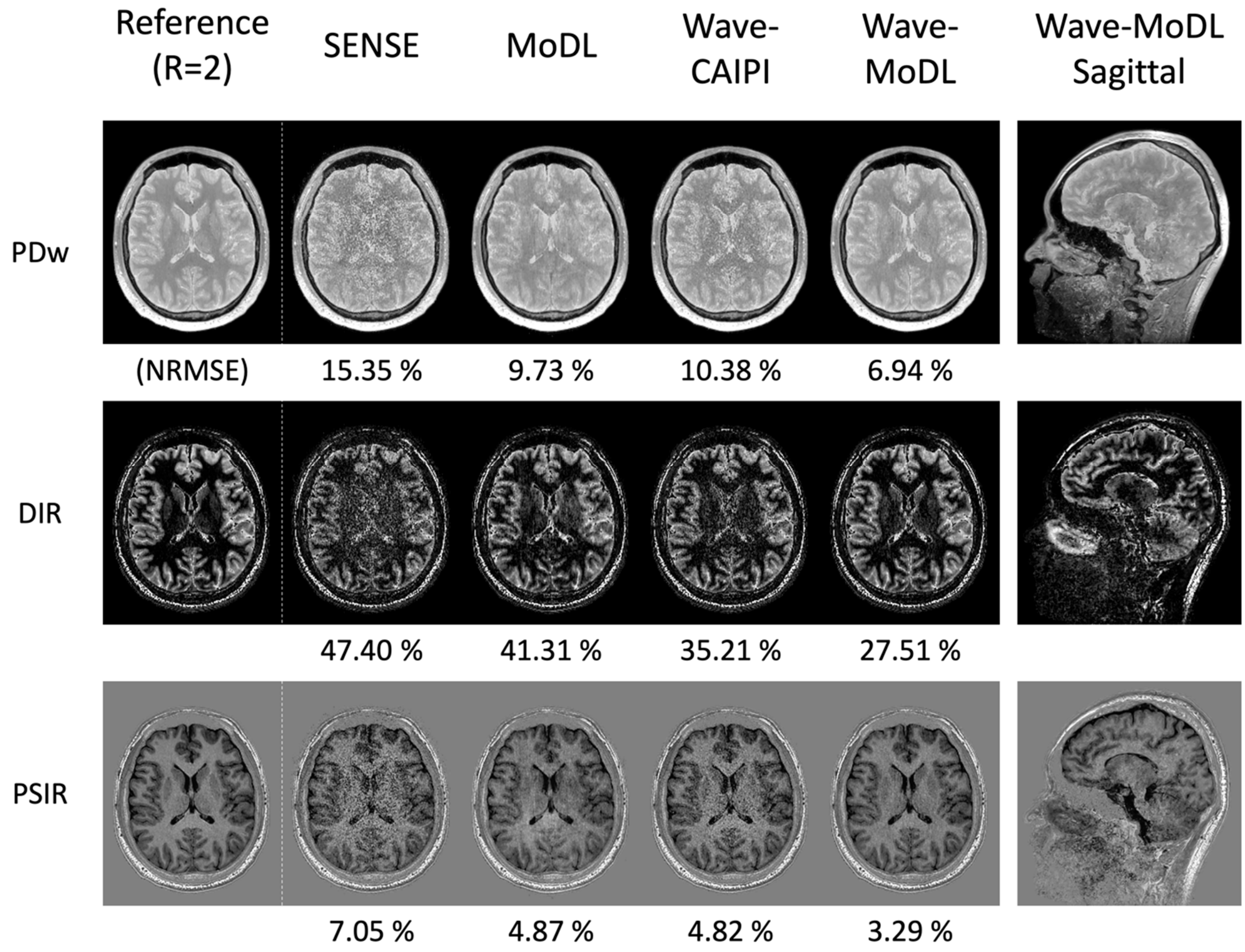

Figure S4. The synthesized double inversion recovery (DIR), and phase-sensitive inversion recovery (PSIR) images at R=4x3-fold acceleration.

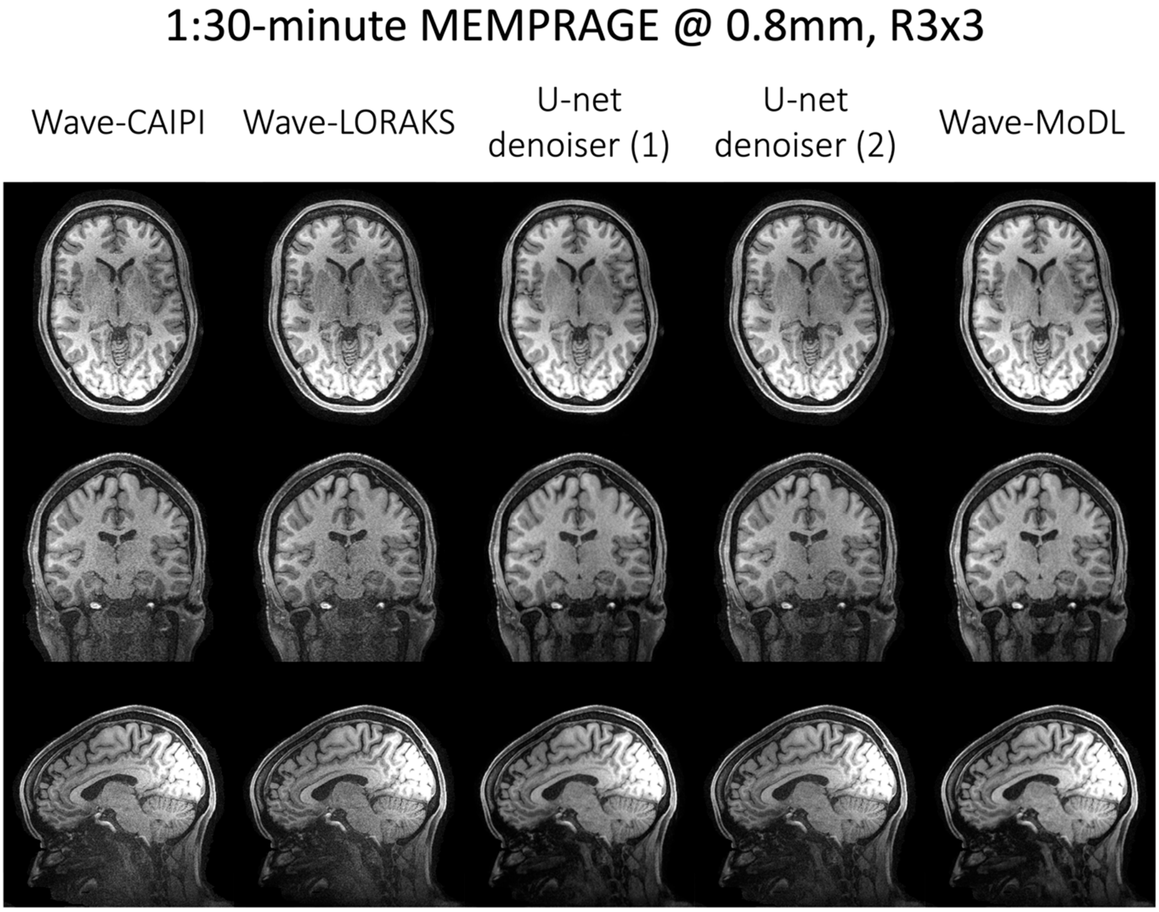

	Wave-CAIPI	Wave-LORAKS	U-net denoiser (1)	U-net denoiser (2)	Wave-MoDL
NRMSE :	16.41 %	13.44 %	9.86 %	11.19 %	11.18 %
SSIM :	0.8453	0.8683	0.7682	0.8235	0.9104
# params :	-	-	34,618,090	105,115	91,458

Figure S5. Wave-CAIPI, wave-LORAKS, U-net denoisers, and wave-MoDL on the MEMPRAGE database at R=3x3-fold and 0.8 mm isotropic voxel resolution. Echo images were combined with root mean squared. NRMSEs and SSIMs were calculated for the entire testing database.
